# Distinct resting-state effective connectivity of large-scale networks in first-episode and recurrent major depression disorder: evidence from the REST-meta-MDD consortium

**DOI:** 10.3389/fnins.2023.1308551

**Published:** 2023-12-11

**Authors:** Yao Zhu, Tianming Huang, Ruolin Li, Qianrong Yang, Chaoyue Zhao, Ming Yang, Bin Lin, the DIRECT Consortium, Xuzhou Li

**Affiliations:** ^1^School of Psychology and Cognitive Science, East China Normal University, Shanghai, China; ^2^Department of General Psychiatry, Shanghai Changning Mental Health Center, Shanghai, China; ^3^Department of Radiology, Children’s Hospital of Philadelphia, Philadelphia, PA, United States; ^4^Faculty of Education, Yunnan Normal University, Kunming, Yunnan, China; ^5^Department of Radiology, The Second Affiliated Hospital, Zhejiang University School of Medicine, Hangzhou, China

**Keywords:** major depressive disorder, first-episode and recurrent, resting-state fMRI, brain networks, effective connectivity

## Abstract

**Introduction:**

Previous studies have shown disrupted effective connectivity in the large-scale brain networks of individuals with major depressive disorder (MDD). However, it is unclear whether these changes differ between first-episode drug-naive MDD (FEDN-MDD) and recurrent MDD (R-MDD).

**Methods:**

This study utilized resting-state fMRI data from 17 sites in the Chinese REST-meta-MDD project, consisting of 839 patients with MDD and 788 normal controls (NCs). All data was preprocessed using a standardized protocol. Then, we performed a granger causality analysis to calculate the effectivity connectivity (EC) within and between brain networks for each participant, and compared the differences between the groups.

**Results:**

Our findings revealed that R-MDD exhibited increased EC in the fronto-parietal network (FPN) and decreased EC in the cerebellum network, while FEDN-MDD demonstrated increased EC from the sensorimotor network (SMN) to the FPN compared with the NCs. Importantly, the two MDD subgroups displayed significant differences in EC within the FPN and between the SMN and visual network. Moreover, the EC from the cingulo-opercular network to the SMN showed a significant negative correlation with the Hamilton Rating Scale for Depression (HAMD) score in the FEDN-MDD group.

**Conclusion:**

These findings suggest that first-episode and recurrent MDD have distinct effects on the effective connectivity in large-scale brain networks, which could be potential neural mechanisms underlying their different clinical manifestations.

## Introduction

Major depressive disorder (MDD) is a prevalent and debilitating psychiatric disorder that affects 4.7% of the global population and is the second leading cause of disability worldwide (Ferrari et al., 2013). Neuroimaging studies have made significant efforts to explore the pathology underlying MDD. Abnormal functional connectivity (FC) within and between large-scale intrinsic brain networks (Yan et al., 2019; Liu et al., 2021; Sun et al., 2022a,b), such as the default mode network (DMN), executive control network (ECN), and salience network (SN), has been found in MDD using resting-state functional magnetic resonance imaging (rs-fMRI). This reflects that the synchronized spontaneous activity among anatomically distinct networks is potentially linked to rumination dysfunction (Hamilton et al., 2015), cognitive impairment (Clark et al., 2009), and emotional dysregulation (Zhao et al., 2021) in patients with MDD. However, inconsistencies in the FC of several networks like DMN, including increases, decreases, both increases and decreases, and no significant changes, have been reported in prior studies of brain networks in MDD (Yan et al., 2019). This may be related to low sensitivity and reliability, as well as limited statistical power due to small sample sizes (Button et al., 2013; Chen et al., 2018), leading to the pathophysiology of MDD remaining unknown.

According to the ICD-10, MDD can be classified as first-episode or recurrent depression (Hiller et al., 1994). The risk of relapse in MDD is directly proportional to the number of episodes (de Jonge et al., 2018). Compared to first-episode MDD, recurrent MDD exhibits more severe depressive and somatic symptoms, greater impairments in verbal memory, executive function, and mental representation processing (Roca et al., 2011; Nigatu et al., 2015), as well as higher medical costs (Kamlet et al., 1995; Biesheuvel-Leliefeld et al., 2012). Therefore, distinguishing the neuropathological mechanisms of first-episode and recurrent MDD is important for developing new and effective treatment protocols. A prior large-sample study found FC reduction of DMN in recurrent but not in first-episode MDD, which was associated with duration of illness rather than medication usage, suggesting this alteration is related to symptom severity (Yan et al., 2019). Another study revealed that compared with healthy controls, both first-episode and recurrent MDD showed reduced FC in the DMN and affective network, whereas the decrease in cognitive control network only occurred in first-episode MDD (Sun et al., 2022a,b). Compared with recurrent MDD, first-episode MDD showed hypoconnectivity in the DMN, dorsal attention network (DAN), and somatomotor network (Liu et al., 2021). However, these FC findings did not consider the direction of information communication between networks.

Effective connectivity (EC) represents the direct or indirect causal effect of one brain region on another (Deshpande et al., 2011; Deshpande and Xiaoping, 2012). In EC methods, Granger causality analysis (GCA) is a relatively data-driven analytical method that does not require the design of a complicated task. It is more convenient for clinical application than model-driven Structural equation modeling (SEM) and Dynamic causal modeling (DCM) (Seminowicz et al., 2004; Schlösser et al., 2008). GCA analyzes the direction of information flow between brain areas using time series of information processing and can depict resting-state directional brain networks (Jiao et al., 2014). A prior study has demonstrated that the EC measure may play a more important role than FC in exploring alterations in disease brains and afford better mechanistic interpretability (Geng et al., 2018). Studies on MDD have reported abnormal EC in several brain regions such as the amygdala (de Almeida et al., 2009), prefrontal cortex (Hamilton et al., 2011), and insula (Iwabuchi et al., 2014; Kandilarova et al., 2018), as well as in networks such as DMN, SN, and DAN (Guo et al., 2020; Li et al., 2020; Wang et al., 2022). However, the similarities and differences of EC between first-episode and recurrent MDD in large-scale networks have been less studied using GCA.

In this study, we obtained resting-state fMRI data from 839 patients with MDD and 788 matched normal controls (NCs) from the Chinese REST-meta-MDD project. We used GCA to explore alterations in the EC within and between brain networks in first-episode and recurrent MDD. We also estimated the correlation between EC and clinical assessments. Our hypothesis was that the two MDD subgroups would show different changes in intra- and inter-network EC.

## Methods

### Participants

We utilized rs-fMRI data from the REST-meta-MDD consortium (Chen et al., 2023), comprising 1,300 MDD patients and 1,128 NCs across 23 sites. Each participant underwent a T1-weighted structural scan and an rs-fMRI scan. The patient inclusion criteria, as reported in the study (Yan et al., 2019), were as follows: (1) 18 years < age < 65 years; (2) education >5 years; (3) fulfillment of the Diagnostic and Statistical Manual of Mental Disorders-IV criteria for MDD; and (4) a total score of ≥8 on the 17-item Hamilton Depression Rating Scale (HAMD) at the time of scanning. The exclusion criteria included: (1) any contraindications for undergoing MRI; (2) poor spatial normalization, coverage, or excessive head motion; (3) incomplete information; and (4) sites with fewer than 10 patients in either group. Consequently, we obtained data from 839 MDD patients and 788 NCs across 17 sites. In terms of subgroups, we compared 227 first-episode drug-naïve (FEDN) patients with 388 matched NCs from five sites, 189 recurrent MDD patients with 423 matched NCs from six sites, and 117 FEDN patients with 72 recurrent MDD patients from two sites. The HAMD and Hamilton Anxiety Rating Scale (HAMA) were employed to assess depression and anxiety symptoms in each patient, respectively.

All data were identified and anonymized. Local Institutional Review Boards approved all contributing studies, and participants signed a written informed consent at each local institution.

### fMRI preprocessing

All rs-fMRI scans were preprocessed at each site utilizing the identical DPARSF protocol as reported in Yan et al. (2019). Specifically, the initial 10 volumes were discarded and slice-timing correction was performed. Subsequently, a rigid body transformation was used to realign the time series of images for each subject. After that, individual T1-weighted images were co-registered to the mean functional image using a 6 degrees-of-freedom linear transformation without re-sampling, and then segmented into gray matter (GM), white matter (WM), and cerebrospinal fluid (CSF). Following this, transformations from individual native space to MNI space were computed using the Diffeomorphic Anatomical Registration Through Exponentiated Lie algebra (DARTEL) tool (Ashburner, 2007) and applied to individual functional images. The Friston 24-parameter model, WM, and CSF signals were removed from normalized data through linear regression. Lastly, a linear trend was included as a regressor to account for drifts in the BOLD signal and temporal band-pass filtering (0.01–0.1 Hz) was applied to all time series.

### Effective connectivity analysis

**Figure 1:**
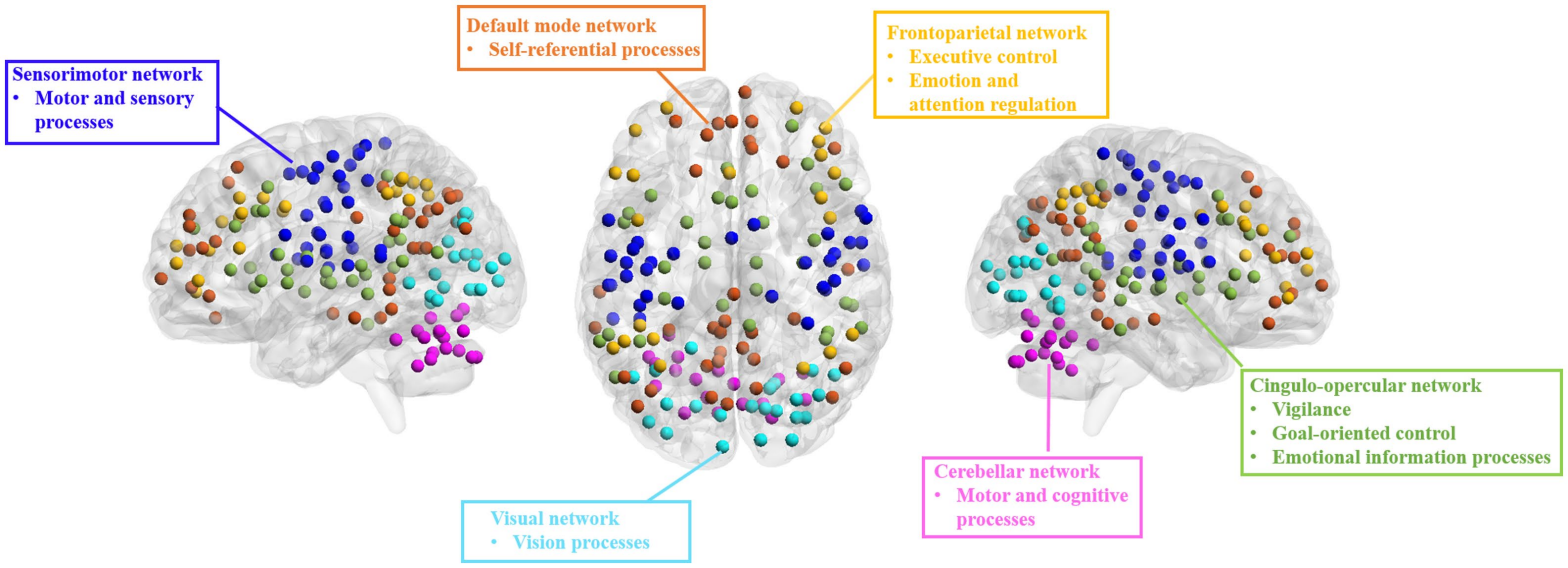
Brain networks from DOS-160 atlas used in the present study. DMN, default mode network; FPN, fronto-parietal network; CON, cingulo-opercular network; SMN, sensorimotor network; VN, visual network; CN, cerebellum network.

We used the DOS-160 atlas (Dosenbach et al., 2010) to segment the brain into 160 regions of interest (ROIs) involved in six networks: cingulo-opercular network (CON), FPN, DMN, sensorimotor network (SMN), visual network (VN), and cerebellum network (CN) (Figure 1). We extracted the averaged time series for each ROI and calculated the EC between any paired ROIs using the GCA method. Then, we computed intra- and inter-network EC by averaging the connectivities between ROIs belonging to the same or different networks, respectively, and the averaged EC with each ROI or network as a seed.

### Statistical analysis

We employed a linear mixed model (LMM) (West et al., 2022) to compare differences in EC between MDD and NC, FEDN and NC, recurrent MDD and NC, and FEDN and recurrent MDD, respectively. The model was following: y ∼ 1 + Diagnosis + Age + Sex + Education + Motion + (1|Site) + (Diagnosis |Site), in which y represents the EC value. This yields *t* and *p* values for the fixed effect of Diagnosis (Yan et al., 2019). To test relationships between EC and clinical assessments, we replaced the ‘*y*’ in the LMM with HAMD or HAMA scores, respectively. The multiple comparisons were corrected using false discovery rate (FDR) correction (*p* < 0.05).

## Results

### Characteristics of participants

As shown in Table 1, two MDD subgroups had no significant differences than NCs in age and gender (*p* > 0.05), but showed lower education than NCs (*p* < 0.001). Recurrent MDD showed a longer duration of illness than FEDN (*p* < 0.001). FEDN and recurrent MDD showed no significant differences in age, gender, and education (*p* > 0.05). Total MDD (mixture of FEDN and recurrent MDD) showed significant differences in education (*p* < 0.001) and gender (*p* = 0.005) but not age (*p* > 0.05) compared with NCs.

**Table 1:** Demographic characteristics of participants.

	MDD vs NC			FEDN vs NC			RMDD vs NC			FEDN vs RMDD		
	MDD (*N* = 848)	NC (*N* = 794)	*p*-value	FEDN (*N* = 232)	NC (*N* = 394)	*p*-value	RMDD (*N* = 189)	NC (*N* = 427)	*p*-value	FEDN (*N* = 119)	RMDD (*N* = 72)	*p*-value
Age	34.3 ± 11.5	34.4 ± 13.0	0.313	32.7 ± 10.4	35.7 ± 14.2	0.154	35.4 ± 12.5	37.1 ± 14.1	0.333	35.4 ± 11.3	36.3 ± 12.7	0.865
Sex (male, *n*%)	474(36.5%)	474(42.1%)	0.005	78(33.6%)	152(38.6%)	0.214	78(41.3%)	167(39.1%)	0.613	40(33.6%)	30(41.7%)	0.263
Education	12.0 ± 3.4	13.6 ± 3.4	<0.001	12.2 ± 3.4	13.6 ± 3.6	<0.001	11.7 ± 3.2	13.4 ± 3.8	<0.001	11.5 ± 3.4	12.0 ± 3.5	0.270
Head Motion	0.07 ± 0.04	0.07 ± 0.04	0.920	0.06 ± 0.03	0.07 ± 0.04	0.455	0.07 ± 0.04	0.07 ± 0.04	0.910	0.06 ± 0.03	0.07 ± 0.03	0.566
Duration (month)	38.4 ± 60.6	NA	NA	17.7 ± 30.8	NA	NA	92.7 ± 86.1	NA	NA	27.0 ± 39.5	88.7 ± 80.1	<0.001
Site	17	17	1.000	5	5	1.000	6	6	1.000	2	2	1.000
HAMD	21.7 ± 6.6	NA	NA	22.5 ± 5.4	NA	NA	17.7 ± 7.8	NA	NA	22.1 ± 4.2	21.3 ± 5.8	0.371
HAMA	19.2 ± 8.9	NA	NA	21.9 ± 9.3	NA	NA	13.9 ± 10.5	NA	NA	11.2 ± 8.3	11.4 ± 10.5	0.342

### Between-group differences in EC of large-scale brain networks

**Figure 2:**
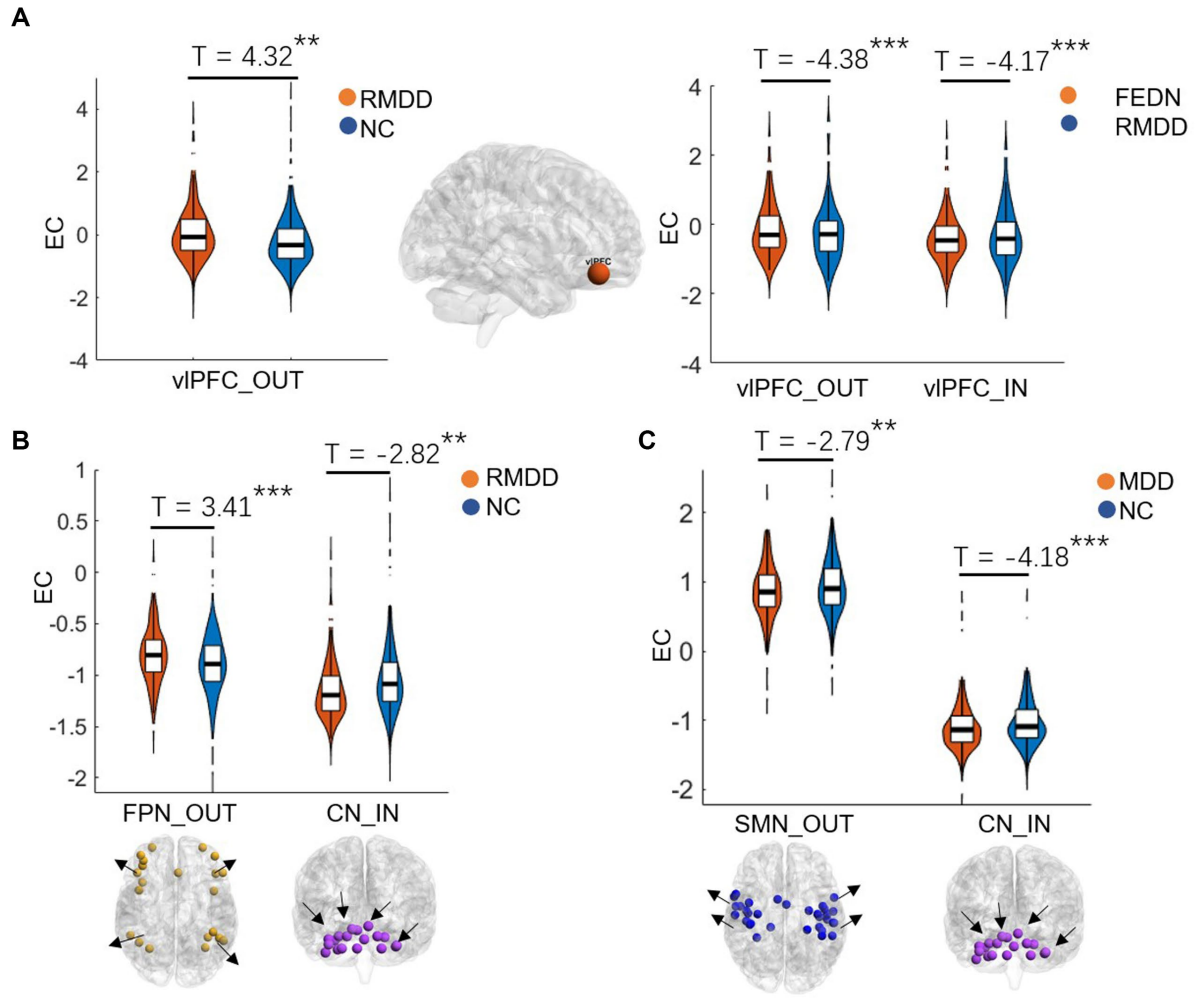
Differences between groups in effective connectivity of brain networks. FEDN, first-episode drug-naïve; RMDD, recurrent major depression disorder; vlPFC, ventral lateral prefrontal cortex; FPN, fronto-parietal network; SMN, sensorimotor network; CN, cerebellum network. **p < 0.05; ***p < 0.001.

**Figure 3:**
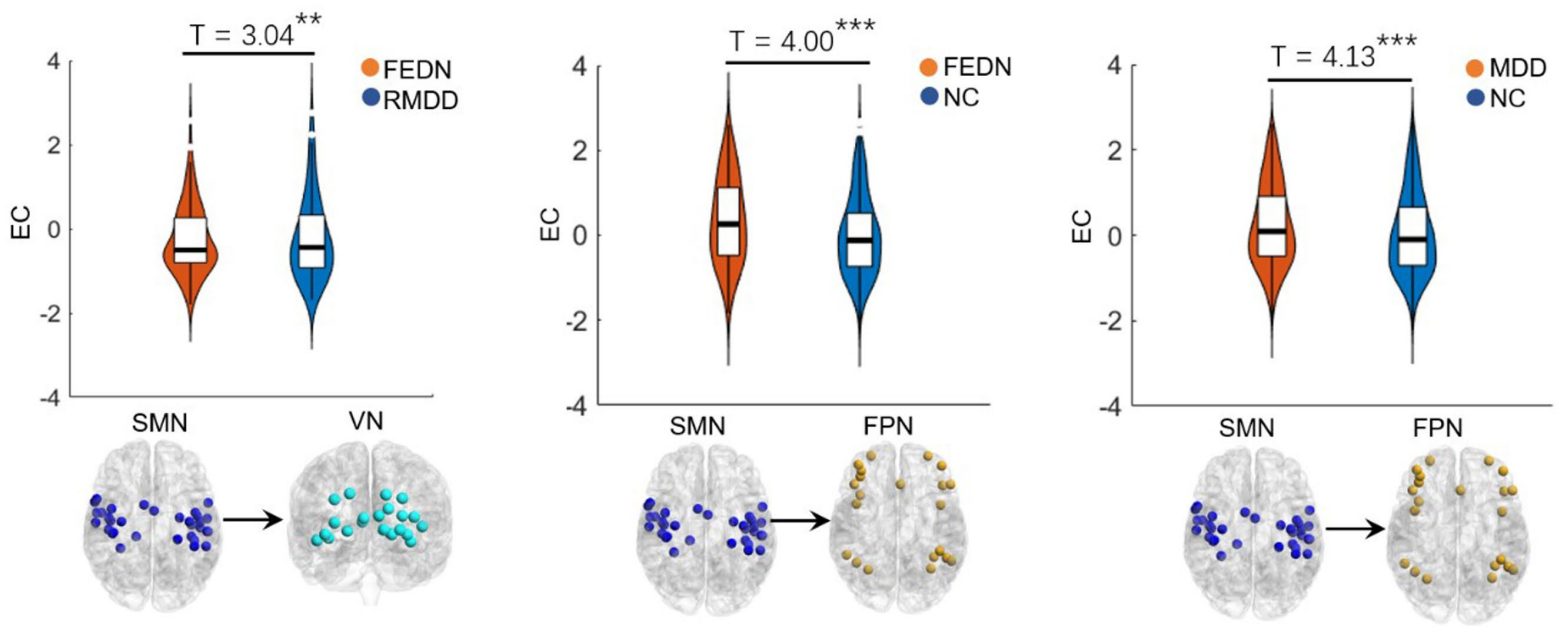
Inter-network differences in effective connectivity between groups. FEDN, first-episode drug-naïve; RMDD, recurrent major depression disorder; FPN, fronto-parietal network; SMN, sensorimotor network; VN, visual network. **p < 0.05; ***p < 0.001.

As shown in Figures 2, 3, total MDD showed decreased afferent EC to the CN, increased efferent EC from the FPN, and increased EC from SMN to FPN compared with NCs. When MDD was divided into two subgroups, FEDN showed increased EC from SMN to FPN compared to NCs, and decreased EC from SMN to VN relative to recurrent MDD. Recurrent MDD showed stronger efferent EC in the ventral lateral prefrontal cortex (vlPFC) with all other regions in the whole brain relative to both NCs and FEDN, and decreased afferent to the CN and efferent EC from the SMN compared with NCs. The FEDN showed no significant difference in seed-based network EC compared to NCs, and the recurrent MDD showed no significant difference in inter-network EC compared to NCs.

### Correlation

**Figure 4:**
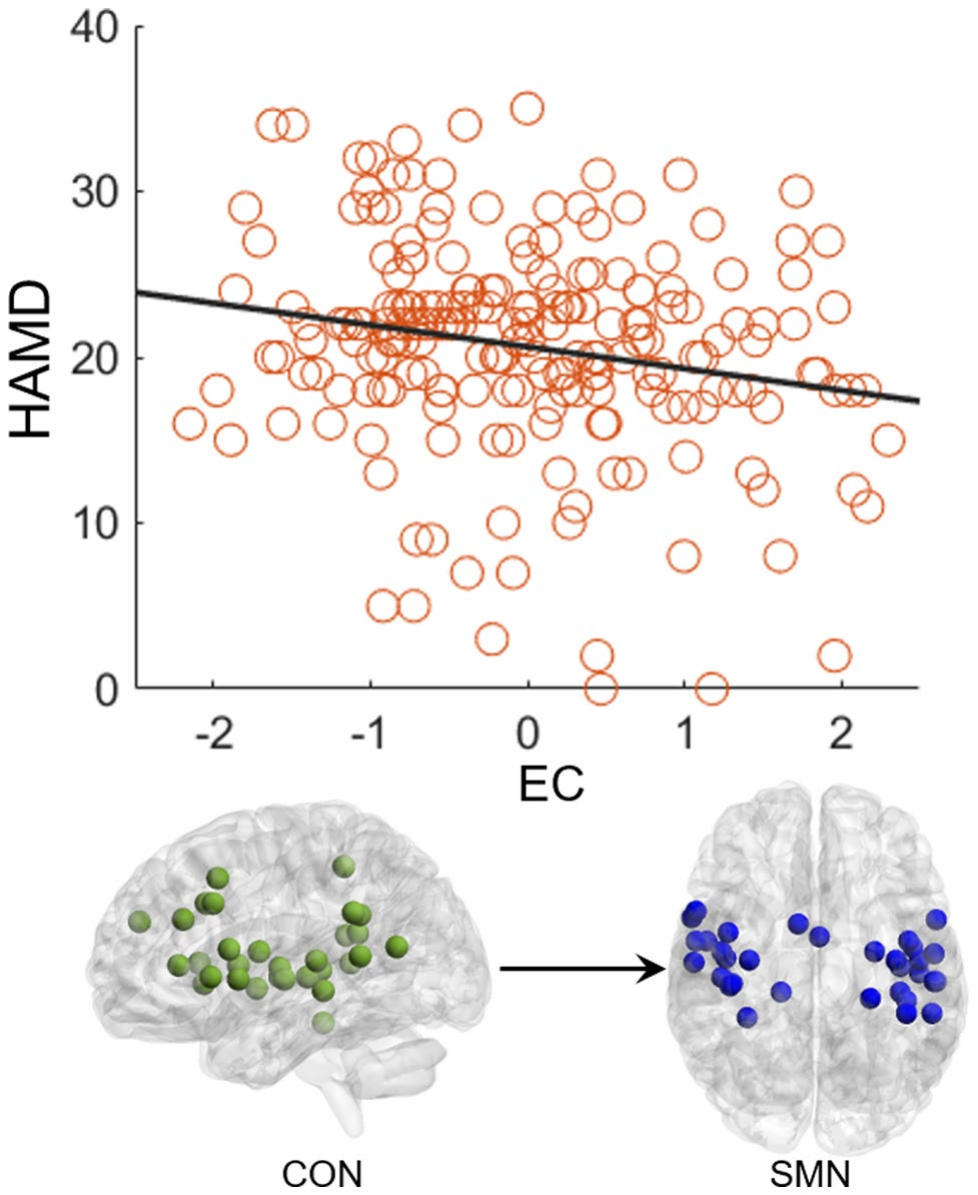
Correlation between effective connectivity and HAMD score. The effective connectivity from CON to SMN shows a significant negative correlation with the HAMD score in the FEDN group.

The EC from CON to SMN showed a significant negative correlation (*p* = 0.004, *R* = −0.20) with the HAMD score in the FEDN group (Figure 4). No significant correlations were observed in the total MDD and recurrent MDD groups. There was no significant correlations between EC and the HAMA score in all groups.

## Discussion

This study used GCA to explore alterations in EC within and between resting-state networks in FEDN and recurrent MDD patients in a large-sample Chinese population. We found that: (1) recurrent and total MDD showed altered EC in the FPN, SMN, and CN compared to NCs, while FEDN and total MDD showed altered inter-network EC from SMN to FPN compared to NCs; (2) two MDD subgroups showed significant differences in intra-network EC of the vlPFC within the FPN and in inter-network EC from SMN to VN; (3) the EC from CON to SMN showed a significant negative correlation with the HAMD score in FEDN but not in recurrent MDD group. These findings suggest that the EC among large-scale brain networks at rest was disrupted in patients with MDD, and that recurrent MDD exhibited different effective connections from FEDN.

The previous studies demonstrated that repetitive transcranial magnetic stimulation to the vlPFC reduced individual depersonalization symptoms (American Psychiatric Association, 2013; Jay et al., 2016). Increased activity in this region is linked with increased fronto-insula/limbic inhibitory regulation (Lemche et al., 2007) and may represent an increased effort to regulate emotions or be indicative of deficits in this area (Langenecker et al., 2007). Compared with healthy controls and MDD patients, bipolar disorder (BD) patients showed increased ventral prefrontal cortical responses to both positive and negative emotional expressions (Lawrence et al., 2004). A prior magnetic resonance spectroscopy study found that recurrent MDD showed more metabolite abnormalities in the ventral frontal cortex compared with both first-episode MDD and controls (Portella et al., 2011). A recent functional near-infrared spectroscopy study demonstrated different neurofunctional activity in frontal regions in FEDN and recurrent MDD, which linked between the level of complexity activation in these regions and cognitive impairment severity of patients (Yang et al., 2023). Another recent fMRI study reported that recurrent MDD had higher spontaneous brain activity in the prefrontal cortex compared to first-episode depression, which showed a positive correlation with depressive symptom severity (Sun et al., 2022a,b). Effective connectivity analysis revealed mutually propagating activation in ventral prefrontal cortex in people with MDD, which predicted higher levels of depressive rumination (Hamilton et al., 2011). Consistently, our study found that recurrent MDD had increased EC in the vlPFC compared with both FEDN and NCs, suggesting more severe depressive symptoms in recurrent patients, possibly associated with depersonalization, emotional regulation, and rumination.

Recent studies have demonstrated that the cerebellum plays a significant role in motor control, cognition, and emotion (Balasubramanian et al., 2021; Su et al., 2021). For example, Liu et al. (2012) found disrupted FC of the CN in adults with major depression, which could be associated with emotional disturbances and cognitive deficits. Liu et al. (2022) reported altered EC of the CN in patients with MDD, which was correlated with deficits in spatial–visual attention and psychomotor disorders. The FPN, also referred to as the executive network, plays a pivotal role in control function, execution, and emotion processing. It is strongly associated with cognitive problems in depression, especially those concerning executive functions. Dysfunctions within the FPN are likely connected to ineffective transmission of information between parietal and prefrontal regions (Brzezicka, 2013). Studies also reported alterations in FC strengths in the frontal and sensorimotor networks (Pang et al., 2020) and disrupted interhemispheric coordination in SMN in MDD patients (Zhang et al., 2023). Moreover, individuals with long-duration MDD showed increased FC in the FPN compared to those with short-duration MDD (Sheng et al., 2022). Similarly, the present study found altered EC of the FPN, CN, and SMN in total MDD and recurrent MDD but not in FEDN, which could be associated with functional impairments of cognitive processing, perception and information integration (Lu et al., 2020), and treatment response-related changes in depression (Dichter et al., 2015). These findings may serve as a potentially effective biomarker for recurrent MDD.

Many studies have found significantly altered connections in low-order networks such as the SMN and VN in MDD patients (Wei et al., 2015; Sambataro et al., 2017). The sensorimotor cortex is a brain region that has attracted much attention in depression research (Ray et al., 2021). Several sensorimotor interventions, including light, music, and physical exercise are known to modulate mood and depressive symptoms (Canbeyli, 2013). Depression gives rise to sensorimotor alterations such as psychomotor retardation or agitation and feelings of fatigue, which are part of the diagnostic criteria for depression (Guha, 2014). Previous studies have found alterations of FC and cerebral blood flow in the SMN related to psychomotor retardation in patients with depression (Yin et al., 2018; Yu et al., 2019), while task-based fMRI studies showed differential reactions of the visual cortex in depression (Rosa et al., 2015; Le et al., 2017). Lu et al. (2020) found reduced between-network FC in auditory and visual networks associated with depression. Kang et al. (2018) demonstrated abnormal primary somatosensory area-thalamic FC in MDD. Moreover, abnormal ECs among the FPN, VN, and SMN networks have been reported to be related to visual attention and cognitive behavior deficits in MDD patients (Kang et al., 2018). Therefore, the present study observed increased EC from SMN to FPN in both total MDD and FEDN compared to NCs, which may be compensation for sensory impairments, psychomotor retardation, and cognitive dysfunction of patients. In addition, a recent study uncovered that the ECs in sensorimotor cortices may serve as a promising and quantifiable candidate marker of depression severity and treatment response (Ray et al., 2021). Another study found that changes in information flow direction from SMN before and after electroconvulsive therapy were significantly correlated with improvement in depressive symptoms in MDD patients (Kyuragi et al., 2023). A small-sample study found that patients with recurrent MDD showed remarkably different effective connections compared to patients with first-episode MDD, especially related to the attention network (Wang et al., 2022). Thus, the increased EC from the SMN to the VN in recurrent MDD relative to FEDN in the present study may be associated with depression severity and treatment of patients. Furthermore, the EC from CON to SMN negatively correlated with the HAMD score may serve as a biomarker to predict the severity of MDD.

### Limitations

The present study has several limitations. First, the correlation analysis relied solely on HAMD scores of depression. There are a large number of rating scales for assessing depression severity, and each with its own advantages and limitations. Thus, the present neuroimaging findings could be further validated with a combination of observer rating scales and objective behavioral measures of depression (Lahnakoski et al., 2020). Second, we were unclear about the medication history of the recurrent MDD patients, and therefore the present findings are in need of replication. Third, MDD patients in the present study were the Chinese populations, which might not be generalized to other regions or populations. Fourth, the use of LMM should be discussed with regard to its potential limitations, such as its comparison to other methods or its applicability to this specific study. Finally, as a cross-sectional study, changes in connections with disease progression cannot be thoroughly reflected by the limited nodes. Further efforts, such as intervention studies with comparisons before and after medication, are required to draw valid conclusions on the impact of EC.

## Conclusion

The present study used the GCA method to investigate differences in EC of large-scale brain networks in FEDN and recurrent MDD patients. We found that recurrent MDD showed altered EC in the FPN, SMN, and CN, while FEDN showed altered inter-network EC from SMN to FPN compared with NCs. Meanwhile, the ECs within FPN and from SMN to VN displayed significant differences between two MDD subgroups. Moreover, the EC from CON to SMN showed a significant negative correlation with HAMD scores in FEDN but not recurrent MDD group. These findings suggest that first-episode and recurrent MDD may have different effective connectivity patterns among large-scale brain networks, which may serve as potential biomarkers for diagnosing MDD.

## Data availability statement

Publicly available datasets were analyzed in this study. This data can be found here: http://rfmri.org/REST-meta-MDD.

## Author contributions

YZ: Writing – original draft, Methodology, Software, Writing – review & editing. TH: Methodology, Writing – original draft. RL: Writing – original draft, Writing – review & editing. QY: Writing – original draft, Writing – review & editing. CZ: Writing – review & editing, Methodology, Software. MY: Writing – review & editing, Methodology, Software. BL: Writing – review & editing, Formal analysis. XL: Writing – review & editing.

## References

[ref1] American Psychiatric Association. (2013). Diagnostic and statistical manual of mental disorders: DSM-5 (American psychiatric association Washington, DC).

[ref2] Ashburner J. (2007). A fast diffeomorphic image registration algorithm. NeuroImage 38, 95–113. doi: 10.1016/j.neuroimage.2007.07.007 17761438

[ref3] Balasubramanian M. Mulkern R. V. Neil J. J. Maier S. E. Polimeni J. R. (2021). Probing in vivo cortical myeloarchitecture in humans via line-scan diffusion acquisitions at 7 T with 250–500 micron radial resolution. Magn. Reson. Med. 85, 390–403. doi: 10.1002/mrm.28419, PMID: 32738088 PMC7951328

[ref4] Biesheuvel-Leliefeld K. E. M. Kersten S. van der Horst H. E. van Schaik A. Bockting C. L. H. Bosmans J. E.. (2012). Cost-effectiveness of nurse-led self-help for recurrent depression in the primary care setting: design of a pragmatic randomised controlled trial. BMC Psychiatry 12, 1–9. doi: 10.1186/1471-244X-12-59 22677092 PMC3403967

[ref5] Brzezicka A. (2013). Integrative deficits in depression and in negative mood states as a result of fronto-parietal network dysfunctions. Acta Neurobiol. Exp. 73, 313–325. PMID: 24129481 10.55782/ane-2013-1939

[ref6] Button K. S. Ioannidis J. P. A. Mokrysz C. Nosek B. A. Flint J. Robinson E. S. J.. (2013). Power failure: why small sample size undermines the reliability of neuroscience. Nat. Rev. Neurosci. 14, 365–376. doi: 10.1038/nrn3475, PMID: 23571845

[ref7] Canbeyli R. (2013). Sensorimotor modulation of mood and depression: in search of an optimal mode of stimulation. Front. Hum. Neurosci. 7:428. doi: 10.3389/fnhum.2013.00428 23908624 PMC3727046

[ref8] Chen X. Bin L. Wang Y.-W. Li X.-Y. Wang Z.-H. Li H.-X.. (2023). The complexity of functional connectivity profiles of the Subgenual anterior cingulate cortex and dorsal lateral prefrontal cortex in major depressive disorder: a DIRECT consortium study. bioRxiv. doi: 10.1101/2023.03.09.531726

[ref9] Chen X. Bin L. Yan C.-G. (2018). Reproducibility of R-fMRI metrics on the impact of different strategies for multiple comparison correction and sample sizes. Hum. Brain Mapp. 39, 300–318. doi: 10.1002/hbm.23843, PMID: 29024299 PMC6866539

[ref10] Clark L. Chamberlain S. R. Sahakian B. J. (2009). Neurocognitive mechanisms in depression: implications for treatment. Annu. Rev. Neurosci. 32, 57–74. doi: 10.1146/annurev.neuro.31.060407.125618 19400725

[ref11] de Almeida J. Cardoso R. Versace A. Mechelli A. Hassel S. Quevedo K.. (2009). Abnormal amygdala-prefrontal effective connectivity to happy faces differentiates bipolar from major depression. Biol. Psychiatry 66, 451–459. doi: 10.1016/j.biopsych.2009.03.024, PMID: 19450794 PMC2740996

[ref12] de Jonge M. Bockting C. L. H. van Oppen P. Van H. L. Peen J. Kikkert M. J.. (2018). The association between the number of previous episodes and modifiable vulnerability factors in remitted patients with recurrent depression. PLoS One 13:e0206495. doi: 10.1371/journal.pone.0206495, PMID: 30388131 PMC6214532

[ref13] Deshpande G. Santhanam P. Xiaoping H. (2011). Instantaneous and causal connectivity in resting state brain networks derived from functional MRI data. NeuroImage 54, 1043–1052. doi: 10.1016/j.neuroimage.2010.09.024, PMID: 20850549 PMC2997120

[ref14] Deshpande G. Xiaoping H. (2012). Investigating effective brain connectivity from fMRI data: past findings and current issues with reference to granger causality analysis. Brain Connect. 2, 235–245. doi: 10.1089/brain.2012.0091, PMID: 23016794 PMC3621319

[ref15] Dichter G. S. Gibbs D. Smoski M. J. (2015). A systematic review of relations between resting-state functional-MRI and treatment response in major depressive disorder. J. Affect. Disord. 172, 8–17. doi: 10.1016/j.jad.2014.09.028, PMID: 25451389 PMC4375066

[ref16] Dosenbach N. U. F. Nardos B. Cohen A. L. Fair D. A. Power J. D. Church J. A.. (2010). Prediction of individual brain maturity using fMRI. Science 329, 1358–1361. doi: 10.1126/science.1194144, PMID: 20829489 PMC3135376

[ref17] Ferrari A. J. Charlson F. J. Norman R. E. Flaxman A. D. Patten S. B. Vos T.. (2013). The epidemiological modelling of major depressive disorder: application for the global burden of disease study 2010. PLoS One 8:e69637. doi: 10.1371/journal.pone.0069637 23922765 PMC3726670

[ref18] Geng X. Junhai X. Liu B. Shi Y. (2018). Multivariate classification of major depressive disorder using the effective connectivity and functional connectivity. Front. Neurosci. 12:38. doi: 10.3389/fnins.2018.00038, PMID: 29515348 PMC5825897

[ref19] Guha Martin. (2014). 'Diagnostic and statistical manual of mental disorders: DSM-5′, Ref. Rev., American Psychiatric Publishing, Inc., Arlington, VA

[ref20] Guo M. Wang T. Zhang Z. Chen N. Li Y. Wang Y.. (2020). Diagnosis of major depressive disorder using whole-brain effective connectivity networks derived from resting-state functional MRI. J. Neural Eng. 17:056038. doi: 10.1088/1741-2552/abbc28, PMID: 32987369

[ref21] Hamilton J. P. Chen G. Thomason M. E. Schwartz M. E. Gotlib I. H. (2011). Investigating neural primacy in major depressive disorder: multivariate granger causality analysis of resting-state fMRI time-series data. Mol. Psychiatry 16, 763–772. doi: 10.1038/mp.2010.46, PMID: 20479758 PMC2925061

[ref22] Hamilton J. P. Farmer M. Fogelman P. Gotlib I. H. (2015). Depressive rumination, the default-mode network, and the dark matter of clinical neuroscience. Biol. Psychiatry 78, 224–230. doi: 10.1016/j.biopsych.2015.02.020, PMID: 25861700 PMC4524294

[ref23] Hiller W. Dichtl G. Hecht H. Hundt W. Mombour W. von Zerssen D. (1994). Evaluating the new ICD-10 categories of depressive episode and recurrent depressive disorder. J. Affect. Disord. 31, 49–60. doi: 10.1016/0165-0327(94)90126-0 8046160

[ref24] Iwabuchi S. J. Peng D. Fang Y. Jiang K. Liddle E. B. Liddle P. F.. (2014). Alterations in effective connectivity anchored on the insula in major depressive disorder. Eur. Neuropsychopharmacol. 24, 1784–1792. doi: 10.1016/j.euroneuro.2014.08.005, PMID: 25219936

[ref25] Jay E.-L. Nestler S. Sierra M. McClelland J. Kekic M. David A. S. (2016). Ventrolateral prefrontal cortex repetitive transcranial magnetic stimulation in the treatment of depersonalization disorder: a consecutive case series. Psychiatry Res. 240, 118–122. doi: 10.1016/j.psychres.2016.04.027, PMID: 27104926 PMC4906152

[ref26] Jiao Z.-Q. Zou L. Cao Y. Qian N. Ma Z.-H. (2014). Effective connectivity analysis of fMRI data based on network motifs. J. Supercomput. 67, 806–819. doi: 10.1007/s11227-013-1010-z

[ref27] Kamlet M. S. Paul N. Greenhouse J. Kupfer D. Frank E. Wade M. (1995). Cost utility analysis of maintenance treatment for recurrent depression. Control. Clin. Trials 16, 17–40. doi: 10.1016/0197-2456(94)00020-4 7743786

[ref28] Kandilarova S. Stoyanov D. Kostianev S. Specht K. (2018). Altered resting state effective connectivity of anterior insula in depression. Front. Psych. 9:83. doi: 10.3389/fpsyt.2018.00083, PMID: 29599728 PMC5862800

[ref29] Kang L. Zhang A. Sun N. Liu P. Yang C. Li G.. (2018). Functional connectivity between the thalamus and the primary somatosensory cortex in major depressive disorder: a resting-state fMRI study. BMC Psychiatry 18, 1–8. doi: 10.1186/s12888-018-1913-6 30340472 PMC6194586

[ref30] Kyuragi Y. Oishi N. Yamasaki S. Hazama M. Miyata J. Shibata M.. (2023). Information flow and dynamic functional connectivity during electroconvulsive therapy in patients with depression. J. Affect. Disord. 328, 141–152. doi: 10.1016/j.jad.2023.02.060 36801417

[ref31] Lahnakoski J. M. Forbes P. A. G. McCall C. Schilbach L. (2020). Unobtrusive tracking of interpersonal orienting and distance predicts the subjective quality of social interactions. R. Soc. Open Sci. 7:191815. doi: 10.1098/rsos.191815, PMID: 32968493 PMC7481680

[ref32] Langenecker S. A. Kennedy S. E. Guidotti L. M. Briceno E. M. Own L. S. Hooven T.. (2007). Frontal and limbic activation during inhibitory control predicts treatment response in major depressive disorder. Biol. Psychiatry 62, 1272–1280. doi: 10.1016/j.biopsych.2007.02.019, PMID: 17585888 PMC2860742

[ref33] Lawrence N. S. Williams A. M. Surguladze S. Giampietro V. Brammer M. J. Andrew C.. (2004). Subcortical and ventral prefrontal cortical neural responses to facial expressions distinguish patients with bipolar disorder and major depression. Biol. Psychiatry 55, 578–587. doi: 10.1016/j.biopsych.2003.11.017 15013826

[ref34] Le T. M. Borghi J. A. Kujawa A. J. Klein D. N. Leung H.-C. (2017). Alterations in visual cortical activation and connectivity with prefrontal cortex during working memory updating in major depressive disorder. NeuroImage Clin. 14, 43–53. doi: 10.1016/j.nicl.2017.01.004, PMID: 28138426 PMC5257188

[ref35] Lemche E. Surguladze S. A. Giampietro V. P. Anilkumar A. Brammer M. J. Sierra M.. (2007). Limbic and prefrontal responses to facial emotion expressions in depersonalization. Neuroreport 18, 473–477. doi: 10.1097/WNR.0b013e328057deb3, PMID: 17496806

[ref36] Li G. Liu Y. Zheng Y. Li D. Liang X. Chen Y.. (2020). Large-scale dynamic causal modeling of major depressive disorder based on resting-state functional magnetic resonance imaging. Hum. Brain Mapp. 41, 865–881. doi: 10.1002/hbm.24845, PMID: 32026598 PMC7268036

[ref37] Liu J. Fan Y. Zeng L.-L. Liu B. Yumeng J. Wang M.. (2021). The neuroprogressive nature of major depressive disorder: evidence from an intrinsic connectome analysis. Transl. Psychiatry 11:102. doi: 10.1038/s41398-021-01227-8, PMID: 33542206 PMC7862649

[ref38] Liu D.-Y. Xuan J. Gao Y. Han J.-F. Li Z. Xi-Wen H.. (2022). From molecular to behavior: higher order occipital cortex in major depressive disorder. Cereb. Cortex 32, 2129–2139. doi: 10.1093/cercor/bhab343, PMID: 34613359 PMC9113303

[ref39] Liu L. Zeng L.-L. Li Y. Ma Q. Li B. Shen H.. (2012). Altered cerebellar functional connectivity with intrinsic connectivity networks in adults with major depressive disorder. PLoS One 7:e39516. doi: 10.1371/journal.pone.0039516, PMID: 22724025 PMC3377654

[ref40] Lu F. Cui Q. Huang X. Li L. Duan X. Chen H.. (2020). Anomalous intrinsic connectivity within and between visual and auditory networks in major depressive disorder. Prog. Neuro-Psychopharmacol. Biol. Psychiatry 100:109889. doi: 10.1016/j.pnpbp.2020.109889, PMID: 32067960

[ref41] Nigatu Y. T. Bültmann U. Sijmen A.%J BMC Public Health Reijneveld (2015). The prospective association between obesity and major depression in the general population: does single or recurrent episode matter? BMC Public Health 15, 1–8. doi: 10.1186/s12889-015-1682-9 25880736 PMC4488044

[ref42] Pang Y. Zhang H. Cui Q. Yang Q. Fengmei L. Chen H.. (2020). Combined static and dynamic functional connectivity signatures differentiating bipolar depression from major depressive disorder. Aust. N. Z. J. Psychiatry 54, 832–842. doi: 10.1177/0004867420924089, PMID: 32456443

[ref43] Portella M. J. de Diego-Adeliño J. Gómez-Ansón B. Morgan-Ferrando R. Vives Y. Puigdemont D.. (2011). Ventromedial prefrontal spectroscopic abnormalities over the course of depression: a comparison among first episode, remitted recurrent and chronic patients. J. Psychiatr. Res. 45, 427–434. doi: 10.1016/j.jpsychires.2010.08.010, PMID: 20875647

[ref44] Ray D. Bezmaternykh D. Mel’nikov M. Friston K. J. Das M. (2021). Altered effective connectivity in sensorimotor cortices is a signature of severity and clinical course in depression. Proc. Natl. Acad. Sci. 118:e2105730118. doi: 10.1073/pnas.2105730118 34593640 PMC8501855

[ref45] Roca M. Armengol S. García-García M. Rodriguez-Bayón A. Ballesta I. Serrano M. J.. (2011). Clinical differences between first and recurrent episodes in depressive patients. Compr. Psychiatry 52, 26–32. doi: 10.1016/j.comppsych.2010.04.011 21220062

[ref46] Rosa M. J. Portugal L. Hahn T. Fallgatter A. J. Garrido M. I. Shawe-Taylor J.. (2015). Sparse network-based models for patient classification using fMRI. NeuroImage 105, 493–506. doi: 10.1016/j.neuroimage.2014.11.021 25463459 PMC4275574

[ref47] Sambataro F. Visintin E. Doerig N. Brakowski J. Holtforth M. G. Seifritz E.. (2017). Altered dynamics of brain connectivity in major depressive disorder at-rest and during task performance. Psychiatry Res. Neuroimaging 259, 1–9. doi: 10.1016/j.pscychresns.2016.11.001 27918910

[ref48] Schlösser R. G. M. Wagner G. Koch K. Dahnke R. Reichenbach J. R. Sauer H. (2008). Fronto-cingulate effective connectivity in major depression: a study with fMRI and dynamic causal modeling. NeuroImage 43, 645–655. doi: 10.1016/j.neuroimage.2008.08.002, PMID: 18761094

[ref49] Seminowicz D. A. Mayberg H. S. McIntosh A. R. Goldapple K. Kennedy S. Segal Z.. (2004). Limbic–frontal circuitry in major depression: a path modeling metanalysis. NeuroImage 22, 409–418. doi: 10.1016/j.neuroimage.2004.01.015, PMID: 15110034

[ref50] Sheng W. Cui Q. Jiang K. Chen Y. Tang Q. Wang C.. (2022). Individual variation in brain network topology is linked to course of illness in major depressive disorder. Cereb. Cortex 32, 5301–5310. doi: 10.1093/cercor/bhac015, PMID: 35152289

[ref51] Su L.-D. Fang-Xiao X. Wang X.-T. Cai X.-Y. Shen Y. (2021). Cerebellar dysfunction, cerebro-cerebellar connectivity and autism spectrum disorders. Neuroscience 462, 320–327. doi: 10.1016/j.neuroscience.2020.05.028, PMID: 32450293

[ref52] Sun J. Chen L. He J. Zhongming D. Ma Y. Wang Z.. (2022a). Altered brain function in first-episode and recurrent depression: a resting-state functional magnetic resonance imaging study. Front. Neurosci. 16:876121. doi: 10.3389/fnins.2022.876121, PMID: 35546875 PMC9083329

[ref53] Sun J. Zhongming D. Ma Y. Chen L. Wang Z. Guo C.. (2022b). Altered functional connectivity in first-episode and recurrent depression: a resting-state functional magnetic resonance imaging study. Front. Neurol. 13:1822. doi: 10.3389/fneur.2022.922207 PMC9475213 36119680

[ref54] Wang Y. Chen X. Liu R. Zhang Z. Zhou J. Feng Y.. (2022). Disrupted effective connectivity of the default, salience and dorsal attention networks in major depressive disorder: a study using spectral dynamic causal modelling of resting-state fMRI. J. Psychiatry Neurosci. 47, E421–E434. doi: 10.1503/jpn.220038

[ref55] Wei M. Qin J. Yan R. Bi K. Liu C. Yao Z.. (2015). Association of resting-state network dysfunction with their dynamics of inter-network interactions in depression. J. Affect. Disord. 174, 527–534. doi: 10.1016/j.jad.2014.12.020, PMID: 25556670

[ref56] West Brady T Welch Kathleen B Galecki Andrzej T. (2022). Linear mixed models: a practical guide using statistical software (Crc Press), Boca Raton, FL.

[ref57] Yan C.-G. Chen X. Li L. Castellanos F. X. Bai T.-J. Bo Q.-J.. (2019). Reduced default mode network functional connectivity in patients with recurrent major depressive disorder. Proc. Natl. Acad. Sci. 116, 9078–9083. doi: 10.1073/pnas.1900390116, PMID: 30979801 PMC6500168

[ref58] Yang T. Wang H. Dai H. Hui J. Zhang J. Li J.. (2023). The fNIRS evaluation of frontal and temporal lobe cortical activation in Chinese first-episode medication-naïve and recurrent depression during a verbal fluency task. Front. Psych. 14:1132666. doi: 10.3389/fpsyt.2023.1132666, PMID: 37113544 PMC10126326

[ref59] Yin Y. Wang M. Wang Z. Xie C. Zhang H. Zhang H.. (2018). Decreased cerebral blood flow in the primary motor cortex in major depressive disorder with psychomotor retardation. Prog. Neuro-Psychopharmacol. Biol. Psychiatry 81, 438–444. doi: 10.1016/j.pnpbp.2017.08.013, PMID: 28823848

[ref60] Yu M. Linn K. A. Shinohara R. T. Oathes D. J. Cook P. A. Duprat R.. (2019). Childhood trauma history is linked to abnormal brain connectivity in major depression. Proc. Natl. Acad. Sci. 116, 8582–8590. doi: 10.1073/pnas.1900801116, PMID: 30962366 PMC6486762

[ref61] Zhang C. Jing H. Yan H. Li X. Liang J. Zhang Q.. (2023). Disrupted interhemispheric coordination of sensory-motor networks and insula in major depressive disorder. Front. Neurosci. 17:1135337. doi: 10.3389/fnins.2023.1135337 36960171 PMC10028102

[ref62] Zhao Y. Zhang F. Zhang W. Chen L. Chen Z. Lui S.. (2021). Decoupling of gray and white matter functional networks in medication-naïve patients with major depressive disorder. J. Magn. Reson. Imaging 53, 742–752. doi: 10.1002/jmri.27392, PMID: 33043540

